# MicroRNA-93 promotes the tumorigenesis of osteosarcoma by targeting TIMP2

**DOI:** 10.1042/BSR20191237

**Published:** 2019-08-23

**Authors:** Hua Zhang, Jidong Zhang, Fanrui Meng, Hanzhong Zhu, Hongyu Yan, Yunliang Guo, Shandi Zhang

**Affiliations:** 1 Orthopaedic Surgery, Chengwu People’s Hospital, Heze 274200, Shandong Province, P.R. China; 2 Medical Department of Qingdao University, Qingdao 266071, Shandong Province, P.R. China; 3 Spinal Surgery Department, Heze Municipal Hospital, Heze 274031, Shandong Province, P.R. China

**Keywords:** EMT, MiR-93, osteosarcoma, TIMP2

## Abstract

Osteosarcoma (OS) is the most frequent primary bone malignancy and affects adolescents and young adults. Recently dysregulation of miRNAs has received more attention because of its extensive role in OS carcinogenesis. This research was designed to verify how microRNA-93 (miR-93) and tissue inhibitor of matrix metalloproteinase 2 (TIMP2) be involved in OS development. At first, the levels of miR-93 and its predictive target gene TIMP2 were detected in OS and osteoblast cell lines, and 62 pairs OS and adjacent non-OS specimens by real-time PCR and western blot. Then, viability, invasion, and epithelial mesenchymal transition (EMT) of OS cell lines were examined when overexpressed or knocked down miR-93, or overexpressed TIMP2. Finally, the interaction between miR-93 and TIMP2 was evaluated using mutation, gain, and loss experiment. Our data indicated that miR-93 was increased while TIMP2 was decreased in both OS cell lines and tissues. MiR-93 high-expression and TIMP2 low-expression were related with poor overall survival and prognosis of OS patients. Overexpression or knockdown experiment indicated that miR-93 enhanced OS cell viability, invasion, and EMT expression. TIMP2 could inhibit OS cell viability, invasion, and EMT expression. Further, miR-93 directly targeted TIMP2 and negatively regulated TIMP2 level in OS cells. And up-regulation of TIMP2 reversed the effects of miR-93 in OS. Finally, miR-93 regulated the oncogenic functions in OS cells by regulating the expression of TIMP2. In conclusion, our study demonstrates that miR-93 may exert an oncogenic function while TIMP2 may act as a tumor suppressor on OS.

## Introduction

Human osteosarcoma (OS) is the most frequent primary bone malignancy and affects adolescents and young adults especially those aged from 15 to 19, and is characterized by occurring at the extremities of long bones and originating from primitive osteogenic mesenchymal cells [1]. Currently, chemotherapeutic treatments combined with surgical methods have extensively applied in treatment of OS [2,3]. Despite considerable progress in the diagnosis and treatment of OS, the metastasis rates and mortality of OS are still very high [4], and the clinical effect of OS treatment remains unsatisfactory [5,6]. Thus, it is particularly important to elucidate study on the molecular mechanism of osteosarcoma.

MicroRNAs (miRNAs) are a kind of regulatory RNAs, which are small endogenous non-coding RNAs, consisting 18–25 nucleotides, and negatively regulate the target gene via binding to its 3′-UTR [7]. Many studies have confirmed that dysregulated miRNAs were contributed to multiple physiological processes in different malignancies (including osteosarcoma), such as apoptosis, proliferation, and autophagy [8–10]. Previous evidence has suggested that miR-93 was abnormally increased in OS patients’ tissues [11]. However, the specific mechanism of miR-93 in osteosarcoma is still obscure. Thence determining the exact molecular mechanisms of miR-93 in OS carcinogenesis might contribute to improving diagnose and prognosis of patients with this tumor.

Tissue inhibitor of matrix metalloproteinase 2 (TIMP2), which is the special member of the TIMP family and can regulate many physiological progression such as tumor growth, metastasis, and angiogenesis through matrix metalloproteinases (MMPs) [12]. There is already established scientific evidence linking the decrease in TIMP2 with the progress of cancers, including ovarian cancer [13], cervical cancer [14], OS [15], however, the specific mechanisms of TIMP2 in OS was still unclear.

Here, we investigated whether miR-93 was associated with OS and the possible mechanism. We demonstrated for the first time that up-regulation of miR-93 and decreasing of TIMP2 are strongly positively correlated with poor prognosis. Overexpression of miR-93 promoted OS cell viability and invasion, and TIMP2 had the opposite effect by functional assays. In addition, regulation of TIMP2 by miR-93 was confirmed in our research on OS, and over-expression of TIMP2 was identified to counteract promoting effect of miR-93 on OS. These newly identified miR-93/TIMP2 axis could regulate proliferation and migration of OS, and represented a potential therapeutic strategy for OS.

## Materials and methods

### Clinical specimens

A total of 62 paired OS specimens and adjacent non-OS specimens were collected from the patients who obtained neither radiotherapy nor chemotherapy in Chengwu People’s Hospital and Heze Municipal Hospital. These specimens were collected between July 2013 and February 2018. After approved by the ethics committee of Chengwu People’s Hospital and Heze Municipal Hospital, we had obtained written informed consent from each patient before collecting the specimens. The demographic features and clinicopathologic data were obtained and organized in Tables 1 and 2. All of the specimens were stored at −80°C for further analysis.

**Table 1:** Association between miR-93 expression and clinicopathological characteristics of patients with OS

Characteristics	Cases	miR-93		*P*-value
		High	Low	
**Age (years)**				0.778
≥18	24	16	8	
<18	38	24	14	
**Gender**				0.731
Female	32	20	12	
Male	30	20	10	
**Tumor size, cm**				0.021*
≤5	32	25	7	
>5	30	15	15	
**TNM stage**				0.012*
I	33	26	7	
II/III	29	14	15	
**Distant metastasis**				0.034*
No	31	24	7	
Yes	31	16	15	
**Location**				0.080
Femur/Tibia	29	22	7	
Elsewhere	33	18	15	

Statistical analyses were performed by the χ^2^ test.

* *P*<0.05 was considered significant.

**Table 2:** Association between TIMP2 expression and clinicopathological characteristics of patients with OS

Characteristics	Cases	TIMP2		*P*-value
		Low	High	
**Age (years)**				0.840
≥18	30	18	12	
<18	32	20	12	
**Gender**				0.059
Female	30	22	8	
Male	32	16	16	
**Tumor size, cm**				0.003*
≤5	34	26	8	
>5	28	12	16	
**TNM stage**				0.029*
I	34	25	9	
II/III	28	13	15	
**Distant metastasis**				0.006*
No	29	23	6	
Yes	33	15	18	
**Location**				0.077
Femur/tibia	32	23	9	
Elsewhere	30	15	15	

Statistical analyses were performed by the χ^2^ test.

* *P*<0.05 was considered significant.

### Cell lines

Human OS cell lines (U-2OS, OS-732, HOS, and Saos-2) and the osteoplastic cell line (hFOB) were obtained from Tianjin Sai’er Biotechnology Co. Ltd. (Tianjin, China). Total cells were incubated in RPMI 1640 medium (Gibco; U.S.A.) seeded with 10% FBS and cultured at 5% CO_2_, 37°C.

### Cell transfection

The miR-93 mimic/inhibitor, negative control vectors (miRNA-NC) and TIMP2 plasmid were acquired from GenePharma (Shanghai; China). OS-732 and U-2OS cells were transfected with TIMP2 plasmid and control plasmid, miR-93 mimic/inhibitor or miRNA-NC through Lipofectamine 3000 (Invitrogen, U.S.A.). The efficiency of transfection was analyzed by RT-qPCR.

### RT-qPCR

RNA was isolated by TRIzol^®^ reagent (Invitrogen; U.S.A.) from OS tissues and cells lines. And cDNA was used to synthesize by PrimeScript RT reagent kit (Takara, China). The results of RT-qPCR were quantified using SYBR Premix Ex TaqII Kit (Takara, China). U6 or GAPDH was used as control to normalize the expression of miR-93 and TIMP2, respectively. The expressions were calculated by 2^−△△ct^ method. Primers are as shown in Table 3.

**Table 3:** Primer sequences for RT-qPCR

Primer		Sequence
miR-93	Forward	5′-AGGCCCAAAGTGCTGTTCGT-3′
	Reverse	5′-GTGCAGGGTCCGAGGT-3′
U6	Forward	5′-CTCGCTTCGGCAGCACA-3′
	Reverse	5′-AACGCTTCACGAATTTGCGT-3′
TIMP2	Forward	5′-GAACATCAACGGGCACCAG-3′
	Reverse	5′-TCCCTCCAGAACCCACAACC-3′
GAPDH	Forward	5′-CTCTGATTTGGTCGTATTGGG-3′
	Reverse	5′-TGGAAGATGGTGATGGGATT-3′

Abbreviations: GAPDH, glyceraldehyde-3-phosphate dehydrogenase; TIMP2, tissue inhibitor of matrix metalloproteinase 2; U6, small nuclear RNA༌snRNA.

### CCK-8 assay

Briefly, 1×10^5^ OS cells were put into 96-well plates. Subsequently, the cells were seeding at 0, 24, 48, and 72 h at 37°C, 5% CO_2_. CCK-8 reagent (10 μl, Dojindo, Japan) was added into each plate. The absorbance was measured using microplate (Bio-Rad, U.S.A.) at 490 nm.

### Transwell assay

The ability of OS cell invasion was examined by a transwell chamber with Matrigel (Clontech, CA). For the migration assay, 5 × 10^3^ OS cells were seeded into the top 24-well transwell chambers while the lower chamber was filled with RPMI 1640 medium supplied with 10% FBS. The cells were incubated in the upper chamber for 24 h at 37°C. Then the invaded cells on the bottom chamber surface were stained with 0.5% crystal violet (Sigma–Aldrich, China) for 10 min at room temperature, respectively. Finally, a microscope (Olympus) was used to obtain cell images and count cell number.

### Target prediction

TargetScan and miRanda were conducted for predicting targets of miR-93. We amplified the binding sites of miR-93 on TIMP2 3′-UTR seed region by PCR.

### Dual luciferase reporter assay

The interconnection between miR-93 and TIMP2 was identified by Luciferase Dual Assay (Promega Corporation, U.S.A.). The wild-type (WT) 3′-UTR or the mutated (MUT) sequence of TIMP2 was cloned into the psiCHECK™-2 vector. The miR-93 mimic/inhibitor, psiCHECK-2-TIMP2-WT or psiCHECK-2-TIMP2-MUT and psiCHECK-2 plasmid were transfected using Lipofectamine 3000 (Invitrogen, U.S.A.). The psiCHECK™-2 vector co-transfected with miR-NC was used as internal control to normalize the transfection efficacy. GloMax fluorescence reader (Promega) was used to detect luciferase value 24 h post-transfection.

### Protein analysis

Radio immune precipitation assay (RIPA) lysis buffer was applied to extract the protein samples. The concentration was quantified by BCA protein assay kit (Thermo, U.S.A.). A 10% SDS-PAGE gels was then separated the proteins (50 μg). Then, protein was transferred to a polyvinylidene fluoride (PVDF) membrane. The PVDF membrane was blocked with 5% non-fatskim milk at room temperature, which was incubated with primary antibodies against TIMP2, epithelial mesenchymal transition (EMT) markers (vimentin, N-cadherin, and E-cadherin), MMP-2, MMP-9 and GAPDH (both from Abcam). After washing, they were probed with secondary antibodies at 37°C for another 2 h. Then, protein immunize activity was observed by ECL (Millipore, U.S.A.). GAPDH acted as internal control. Protein expression was examined using a Bio-Spectrum Imaging System (UVP, LLC Upland, CA, U.S.A.).

### Statistical analysis

Our statistical analysis was carried out applying GraphPad 7.0 (GraphPad Software Inc., U.S.A.). The data were expressed as mean ± standard deviation (SD). The *P*-values were analyzed through Student’s *t*-test and one-way ANOVA. Overall survival rates were analyzed through Kaplan–Meier method. *P*<0.05 was considered statistically significant.

## Results

### TIMP2 was lowly expressed and negatively correlated with miR-93 in OS

**Figure 1:**
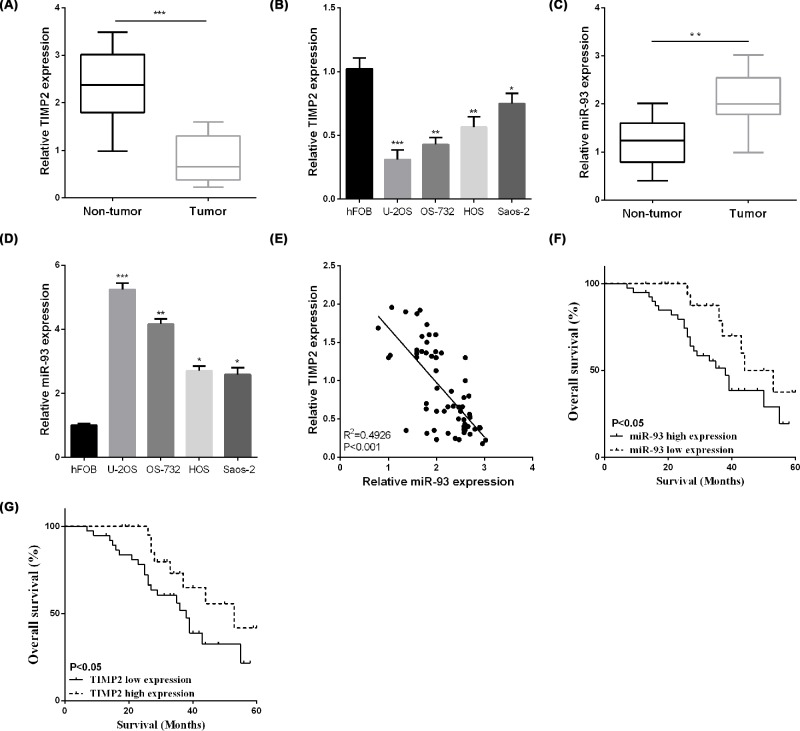
TIMP2 was lowly expressed and negatively correlated with miR-93 in OS (**A,B**) The TIMP2 expression was detected by RT-qPCR in OS tissues and cells. (**C,D**) The expression of miR-93 was detected by RT-qPCR in OS tissues and cells. (**E**) MiR-93 negatively related to TIMP2. (**F,G**) Kaplan–Meier curve in patients with OS. *P<0.05, **P<0.01.

First, we detect the expression of miR-93 / TIMP2 in OS tissues and cell lines. The TIMP2 relative expression was reduced in OS tissues as presented in Figure 1A. And then, we detected the TIMP2 mRNA level in OS cell lines, it showed that TIMP2 mRNA level was also lower in OS cells compare to the normal hFOB cells (Figure 1B). The miR-93 expression was significantly enhanced in OS tissues in comparison with the paratumor tissues (Figure 1C), which was opposite to the TIMP2 expression. As demonstrated in Figure 1D, the expression of miR-93 was also enhanced in OS cell lines than hFOB cells. According to the results of Figure 1B,D, the OS-732 and U-2OS present the highest level of miR-93 and the lowest level of TIMP-2, so we chose these two cell lines for the further studies. And interestingly, statistical analysis demonstrated that miR-93 was negatively related to TIMP2 in OS clinical specimens as presented in Figure 1E.

### The prognostic value and clinicopathological features of miR-93 and TIMP2 for patients with OS

Kaplan–Meier analysis indicated that OS patients with high miR-93 expression have worse overall survival (Figure 1F). Furthermore, the same tendency was obtained in low TIMP2 expression group (Figure 1G). These above results suggested that miR-93/TIMP2 axis was closely connected with the OS pathogenesis. To better determine the clinical significance of miR-93/TIMP2 axis in OS, the median expression level of miR-93/TIMP2 axis was defined as a cut-off value to divide different subgroups named low and high group, respectively. As demonstrated in Tables 1 and 2, the expression of miR-93 (Table 1) and TIMP2 (Table 2) were notably correlated with tumor size, TNM stage, and distant metastasis.

### MiR-93 promoted the cell viability, invasion, and EMT of OS

**Figure 2:**
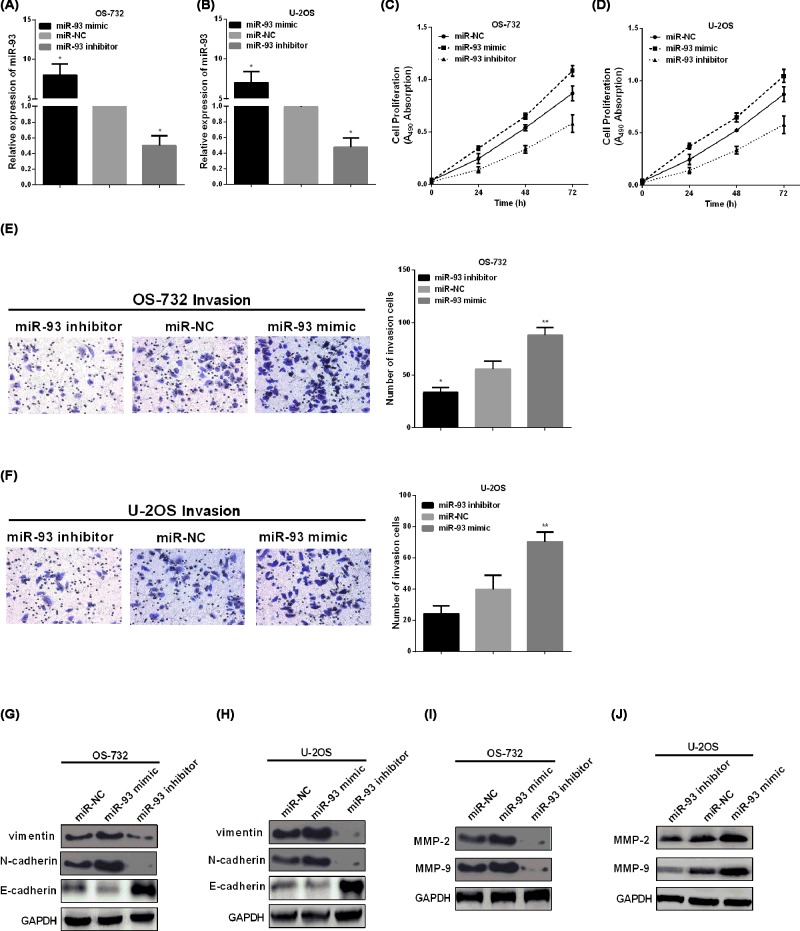
MiR-93 promoted the cell viability, invasion, and EMT of OS (**A,B**) MiR-93 expression regulated by miR-93 mimic/inhibitor was examined in OS cells. (**C,D**) MiR-93 mimic/inhibitor regulated OS cell proliferation. (**E,F**) Cell invasion was regulated by miR-93 mimic/inhibitor in OS. (**G,H**) The protein levels of E-cadherin, vimentin, and N-cadherin in OS-732 and U2-OS. (**I,J**) The protein levels of MMP-2 and MMP-9 in OS-732 and U2-OS. *P<0.05, **P<0.01, ***P<0.001.

Gain- or loss-of-function experiment was performed to confirm the regulatory roles of miR-93 in OS cells *in vitro*. In Figure 2A,B, we can see that miR-93 mimic significantly enhanced its expression while miR-93 inhibitor reduced the expression of miR-93. As CCK-8 results showed that miR-93 overexpression significantly increased the cell viability versus to the miR-NC group, while down-regulation of miR-93 resulting in suppressing OS cell viability as presented in Figure 2C,D. Furthermore, the invaded cells were prominently raised after transfecting with miR-93 mimic and reduced by miR-93 inhibitor (Figure 2E,F). The results of western blotting indicated that enforced up-regulation of miR-93 enhanced the expression of vimentin and N-cadherin, whereas reduced E-cadherin expression, while miR-93 inhibitor has the opposite effect (Figure 2G,H). Previous studies suggested that matrix metalloproteinase (MMP) play important roles in the migration and invasion of OS [16]. To investigate miR-93 could regulate the related genes, western blot showed that miR-93 overexpression significantly increased the MMP-2 and MMP-9 expression, however, miR-93 knockdown obviously decreased the MMP-2 and MMP-9 expression (Figure 2I,J).

### TIMP2 inhibited the cell viability and invasion of OS

**Figure 3:**
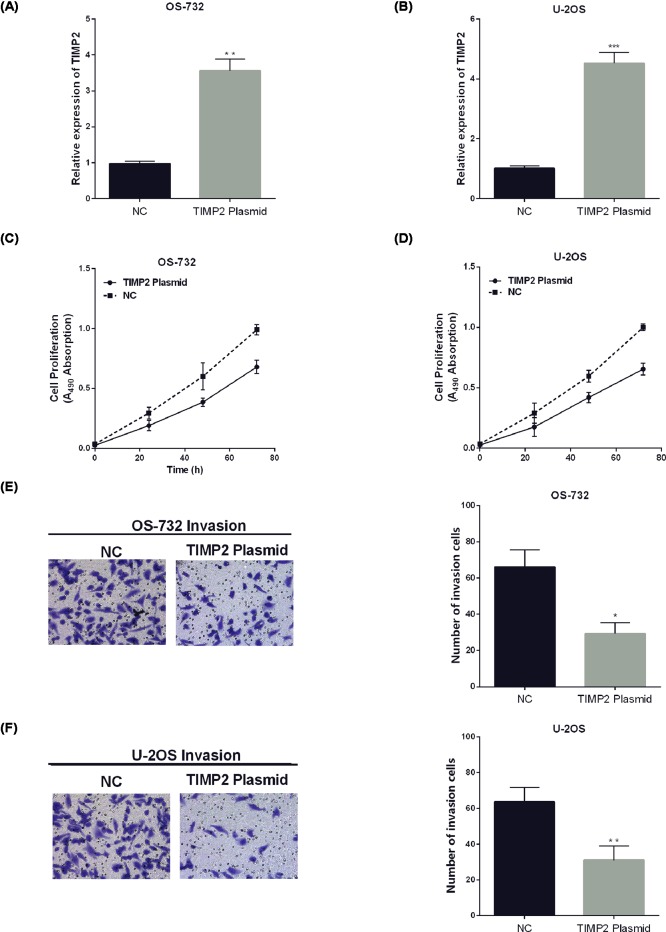
TIMP2 inhibited cell viability and invasion of OS (**A,B**) Expression of TIMP2 in OS-732 and U-2OS cells were detected by RT-qPCR. (**C,D**) TIMP2 plasmid regulated OS cell proliferation. (**E,F**) Cell invasion was regulated by TIMP2 plasmid in OS.

Since TIMP2 mRNA was dramatically reduced in OS patients’ specimens and cell lines, we next investigated the effect of TIMP2 on the progression of OS. We first up-regulated the TIMP2 expression in OS-732 and U-2OS cells by using TIMP2-overexpressing plasmid. The efficiency of TIMP2-overexpressing plasmids was detected by RT-qPCR (Figure 3A,B). Then, CCK-8 was chosen to detect the influence of TIMP2-overexpressing on OS proliferation. CCK-8 data showed that TIMP2-overexpression significantly inhibited OS-732 and U-2OS cell viability compared with control groups (Figure 3C,D). Moreover, the invaded cells were prominently reduced after transfecting with TIMP2-overexpressing plasmid in comparison with control groups as presented in Figure 3E,F.

### TIMP2 was a direct target of miR-93

**Figure 4:**
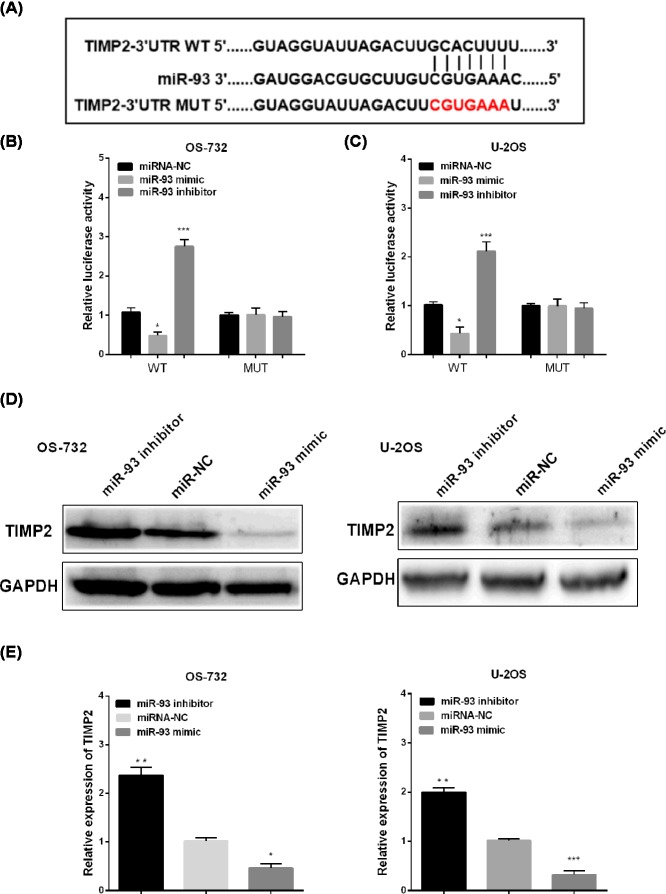
TIMP2 was target of miR-93 (**A**) TIMP2 had binding sites with miR-93. (**B,C**) Luciferase assay detected the role of miR-93 in OS cells. (**D,E**) The expression of TIMP2 was observed in OS cells. *P<0.05, **P<0.01.

Here, we investigated correlation between miR-93 and TIMP2 in OS. Bioinformatics analysis was applying to predict the binding sites, and the binding sites was shown in Figure 4A. The luciferase assay showed that miR-93 overexpression notably reduced TIMP2-WT luciferase activity in OS cells, but had no influence on TIMP2-MUT (Figure 4B,C). Whereas, the TIMP2 protein level or mRNA level were significantly suppressed through overexpressing miR-93, while miR-93 inhibitor notably enhanced the mRNA and protein levels of TIMP2 as shown in Figure 4D,E.

### MiR-93 promoted OS cell viability and invasion via TIMP2

**Figure 5:**
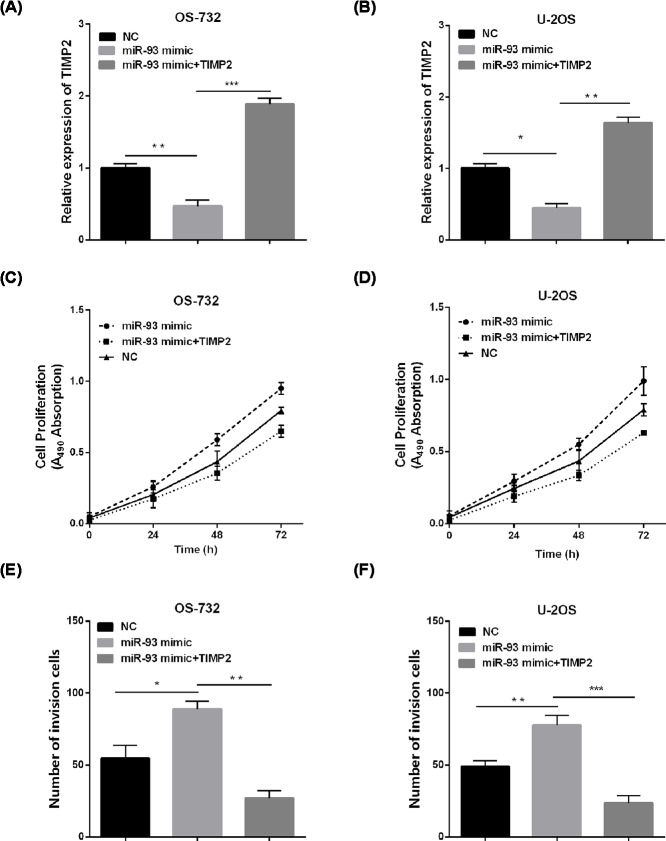
miR-93 promoted OS cell viability and invasion by targeting TIMP2 (**A,B**) Western blotting was used to detect TIMP2 expression after transfection with TIMP2. (**C,D**) Cell viability was detected via CCK-8. (**E,F**) Transwell assays were performed to measure the invasion. *P<0.05, **P<0.01, ***P<0.001.

To further explore the regulation of TIMP2 in miR-93-mediated effect on OS cells, the efficiency of TIMP2-overexpressing plasmid and miR-93 mimic was first analyzed by RT-qPCR in OS-732 and U-2OS cells (Figure 5A,B). CCK-8 assay and invasion assay demonstrated that miR-93 mimic could promote the cell viability, invasion of OS cells as previous results shown, up-regulation of TIMP2 could rescue the cell viability promotion of OS cells resulted from the miR-93 mimic (Figure 5C,D). Besides, the same effects were obtained on the invasion capacity of OS cells (Figure 5E,F). In all, these data suggested that TIMP2 was involved in miR-93-mediated regulation.

## Discussion

OS is the most frequent primary bone malignancy, affects adolescents and young adults, and is characterized by high incidence, great potential for metastasis and rapid progression [17,18]. Although years of continuous progress have been obtained in OS therapy, the metastasis rates and mortality of OS are still very high, and the clinical effect of OS treatment remains unsatisfactory [19]. The molecular mechanisms of OS carcinogenesis, especially regarding alterations of miRNAs, have attracted much attention in recent decades.

Recently increasing evidences indicated miRNAs was important for different malignancies, and many miRNAs were dysregulated in human malignancies, including OS [20,21]. MiR-93 can find dysregulated in multiple tumors, such as hepatocellular carcinoma [22], prostate cancer [23], bladder cancer [24]. However, the effect of miR-93 on OS cell remains unknown. As a part of our ongoing effort for the identification of natural products with anti-cancer effects, we present here first time that miR-93 up-regulated in OS tissues and cell line, directly repressing TIMP2 expression. Its high-expression was obviously associated with adverse clinical pathological issues of OS patients, including tumor size, TNM stage and clinical stage. Moreover, the patients with high miR-93 had a worse overall survival. MiR-93 promoted cell viability, invasion, MMPs, and EMT while miR-93 knockdown has the opposite finding. These data suggest that miR-93 plays a critical role in OS prognosis.

This study also suggested TIMP2 was a candidate target of miR-93 in OS. As an endogenous inhibitor of MMPs, TIMP2 was decreased in various types of cancer, including ovarian cancer [13], cervical cancer [14], and OS [15]. Thence, it attracted our attention to investigate whether TIMP2 was a mediator for miR-93 tumor promotor role in OS cells. Especially, a recent published article indicated that overexpression of TIMP2 could inhibit cell [15], but the underlying mechanisms in OS-732 and U-2OS cells are not fully explored. We accepted the analogous results in OS-732 and U-2OS cells with this. Low expression of TIMP2 was obtained in OS tissues and cells, and miR-93 was negatively related to TIMP2 in OS tissues. Luciferase reporter and western blotting also showed over-expression of miR-93 inhibited TIMP2 expression. Previous study showed TIMP2 was direct target of miR-93 [25]. Furthermore, the overall survival of TIMP2 low expression group was significantly worse verse to high TIMP2 expression group. The above findings suggested miR-93 may act as tumor oncogene by increasing TIMP2 expression in OS progression. And the TIMP2 over-expression reverses the promotional effects of miR-93 in OS cells.

## Conclusion

In this study, the results of the present study suggest that miR-93 was a tumor activator in human OS via directly regulating TIMP2. Functional studies showed that miR-93 inhibited and TIMP2 promoted cell viability and invasion in OS. Additionally, we identified that TIMP2 was identified as direct target of miR-93, and which mediates its promoted function in osteosarcoma cells. Collectively, miR-93/MMPs/TIMP2 pathway may be new biomarkers for prognostic prediction and novel therapeutic promising target for OS. Further studies to look for their candidate value in other tumors are still needed.

## Abbreviations

EMT: epithelial mesenchymal transition
miRNA: microRNA
MMP: matrix metalloproteinase
OS: osteosarcoma
PVDF: polyvinylidene fluoride
TIMP2: tissue inhibitor of matrix metalloproteinase 2
