# Expression of Concern: MicroRNA-93 promotes the tumorigenesis of osteosarcoma by targeting TIMP2

**DOI:** 10.1042/BSR20191237_EOC

**Published:** 2026-09-17

**Authors:** Hua Zhang, Jidong Zhang, Fanrui Meng, Hanzhong Zhu, Hongyu Yan, Yunliang Guo, Shandi Zhang

**Affiliations:** 1 Orthopaedic Surgery, Chengwu People's Hospital, Heze 274200, Shandong Province, P.R. China; 2 Medical Department of Qingdao University, Qingdao 266071, Shandong Province, P.R. China; 3 Spinal Surgery Department, Heze Municipal Hospital, Heze 274031, Shandong Province, P.R. China

**Keywords:** EMT, MiR-93, osteosarcoma, TIMP2

*Bioscience Reports* has been made aware of potential issues surrounding the scientific validity of this paper and is hence issuing a permanent Expression of Concern to notify readers. A duplication has been identified between the Figure 2H N-cadherin bands and the Figure 2I MMP-2 bands. The authors were contacted regarding the similarity but did not respond. As such, the article will remain published but with an associated Expression of Concern.

